# Long term survival following cryoablation with adjuvant Toripalimab for anorectal malignant melanoma: a case report

**DOI:** 10.3389/fonc.2025.1465645

**Published:** 2025-01-24

**Authors:** Xuejun Jiang, Zujin Ji, Peng Li, Fangjun Yuan, Xinyi Lei, Yong Yang

**Affiliations:** ^1^ Department of Colorectal and Anal Surgery, Sinopharm Dongfeng General Hospital, Hubei University of Medicine, Shiyan, Hubei, China; ^2^ Department of Medical Ultrasonics, Sinopharm Dongfeng General Hospital, Hubei University of Medicine, Shiyan, Hubei, China; ^3^ Department of General Surgery, Sinopharm Dongfeng General Hospital, Hubei University of Medicine, Shiyan, Hubei, China

**Keywords:** anorectal malignant melanoma, cryosurgery, sphincter preservation, case report, Toripalimab, PD-1

## Abstract

**Background:**

Anorectal malignant melanoma is a rare subtype of melanoma with a poor prognosis. Despite this, some patients decline Miles’ operation due to the sigmoid colostomies that follow abdominoperineal resections in cases of anorectal malignant melanoma.

**Case report:**

We report the case of an 80-year-old woman diagnosed with anorectal malignant melanoma who underwent cryosurgery accompanied by adjuvant PD-1 therapy to maintain anal sphincter function. The results indicated that we successfully achieved the goal of sphincter preservation and therapeutic efficacy. The patient derived significant benefits from the cryoablation treatment.

**Conclusions:**

Ultrasound-guided trans-anal cryoablation, when combined with adjuvant PD-1 therapy, offers a novel treatment approach for patients with anorectal malignant melanoma. Our results have confirmed the advantages of this treatment regimen, particularly for those desiring to retain anal sphincter function. Further studies are required to substantiate the efficacy of ultrasound-guided trans-anal cryoablation with adjuvant Toripalimab and to elucidate its underlying mechanisms.

## Introduction

Anorectal malignant melanoma (ARMM) is a rare subtype of melanoma that differs from cutaneous melanoma, and its incidence continues to rise (1). Patients with ARMM often face the dilemma of late diagnosis, which can result in a poor prognosis due to the absence of early and typical clinical signs (2). There is ongoing debate regarding the therapeutic approach for ARMM; however, surgical excisions, including wide local excision and abdominoperineal resection, are considered the optimal treatment for ARMM (3, 4). It has been reported that immune checkpoint inhibitors have been utilized in the treatment of ARMM (5, 6). Additionally, in patients with liver metastatic melanoma undergoing PD-1 inhibitor therapy, cryotherapy has been applied for the treatment of liver metastases (7). The median survival period for ARMM is 33 months for localized diseases, 18 months for regional diseases, and 6 months for distant diseases, respectively (8). Nevertheless, some patients require Miles’ operations, but a portion of them decline due to the sigmoid colostomies that follow abdominoperineal resections.

Cryotherapy, as a form of local ablation therapy, has been extensively used in the treatment of various tumors, such as pancreatic cancer, renal cell carcinoma, and hepatocellular carcinoma (9–11). In our center, cryoablation has been employed in the treatment of rectal cancer (12, 13), offering advantages such as minimal anesthesia risk, reduced surgical risk, rapid recovery, the potential for repeat treatments, and most importantly, the possibility of preserving the anus. Herein, we present a novel treatment strategy for a patient with ARMM aimed at preserving the anal sphincter and enhancing the quality of life.

## Case presentation

**Figure 1:**
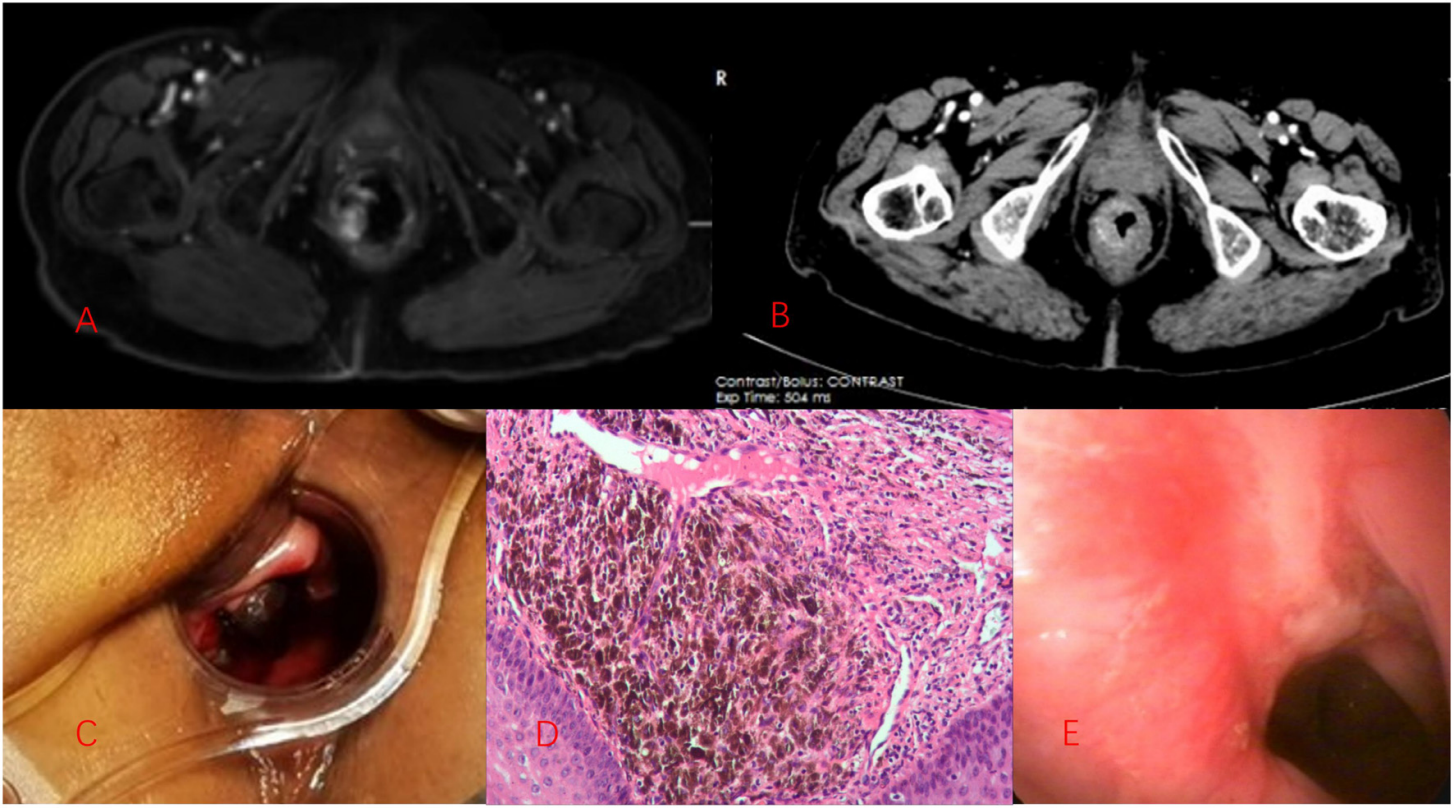
Images of the patient’s examination. **(A)** illustrates the ARMM as seen on MRI. **(B)** depicts the enhancement computed tomography imaging, which revealed thickening of the lower rectal wall, with no evidence of peripheral or distant metastasis. **(C)** shows that anoscopy detected a black mass situated at the dentate line, measuring 2.5 × 1.5 cm. **(D)** presents the immunohistochemical findings: S-100 (+), HMB45 (+), Melan-A (+), Ki-67 (positive rate of 50%), EGFR (+/–). **(E)** is an endoscopic image taken 3 months post-cryoablation.

The patient was an 80-year-old woman experiencing recent intermittent dyschezia and painless rectal bleeding. She had multiple comorbidities, including diabetes, hypertension, and coronary heart disease, which were being managed with conventional medication. A digital rectal examination revealed a black, rubbery-textured mass protruding from the anal verge, resembling a nevus on the right anterior wall of the rectum. Anoscopy disclosed a black mass situated at the dentate line, measuring 2.5 × 1.5 cm (**Figure 1C**). Pelvic magnetic resonance imaging (MRI) indicated a thickened rectal wall with well-defined structures surrounding the intestinal canal. The upper rectum appeared dilated, and clinical considerations pointed towards melanoma (Stage I, according to the Slingluff staging system) (**Figure 1A**). Contrast-enhanced computed tomography (CT) imaging demonstrated thickening of the lower rectal wall, with no evidence of peripheral or distant metastasis (**Figure 1B**). Tumor biomarkers were found to be within the normal range.

The biopsy specimen from the tumor was diagnosed as malignant melanoma, with an ICD-O code of 8720/3. Immunohistochemical analysis yielded the following results: S-100 (+), HMB45 (+), Melan-A (+), Ki-67 (positive rate of 50%), and EGFR (+/–) (refer to **Figure 1D**). No mutations in CKIT, BRAF, or NRAS were detected. Based on the preoperative examination findings, the tumor was classified as Stage I according to the Slingluff staging system.

## Pre-cryoablation preparation

The multidisciplinary team (MDT) tended to radical resection after a discussion. The patient was considered ineligible for abdominal perineal resection (APR) for fear of enterostomy. Therefore, the MDT suggested cryoablation with adjuvant Toripalimab can be an alternative regimen for the patient. She followed our advice and chose cryoablation after (MDT) discussions. Interrupting oral aspirin before cryoablation was necessary to avoid bleeding. An enema with 0.9% sodium chloride solution was given to clean the rectum the day before cryosurgery. Injecting antibiotic to prevent perirectal infection half an hour before cryosurgery.

## Refrigeration equipment

APCA1 - type 3 (Registration No.: GYGX (Z) Zi 2003 No. 23580109) cryoablation system served as refrigeration equipment to perform cryoablation in our center. The freezing medium was liquid nitrogen.

## Cryoablation intervention

The detailed cryoablation process is listed below. The specific ablation depth and range can be achieved through ultrasound (US) monitoring.

Step 1: Exposing the tumor after sacral anesthesia takes effect.
Step 2: Pressing the cryoprobe toward the tumor center.
Step 3: Switching on the refrigeration.
Step 4: Adjusting the output pressure to 0.3 KPa and maintaining freezing.
Step 5: Waiting for the ice ball to cover the tumor.
Step 6: Switching off refrigeration until the ice ball was left away from the nearest tumor edge by 5 mm, which was monitored by the US (took approximately 4.5 min).
Step 7: Waiting for passive thawing.
Step 8: Repeating the above steps twice.

## Intraoperative image display

**Figure 2:**
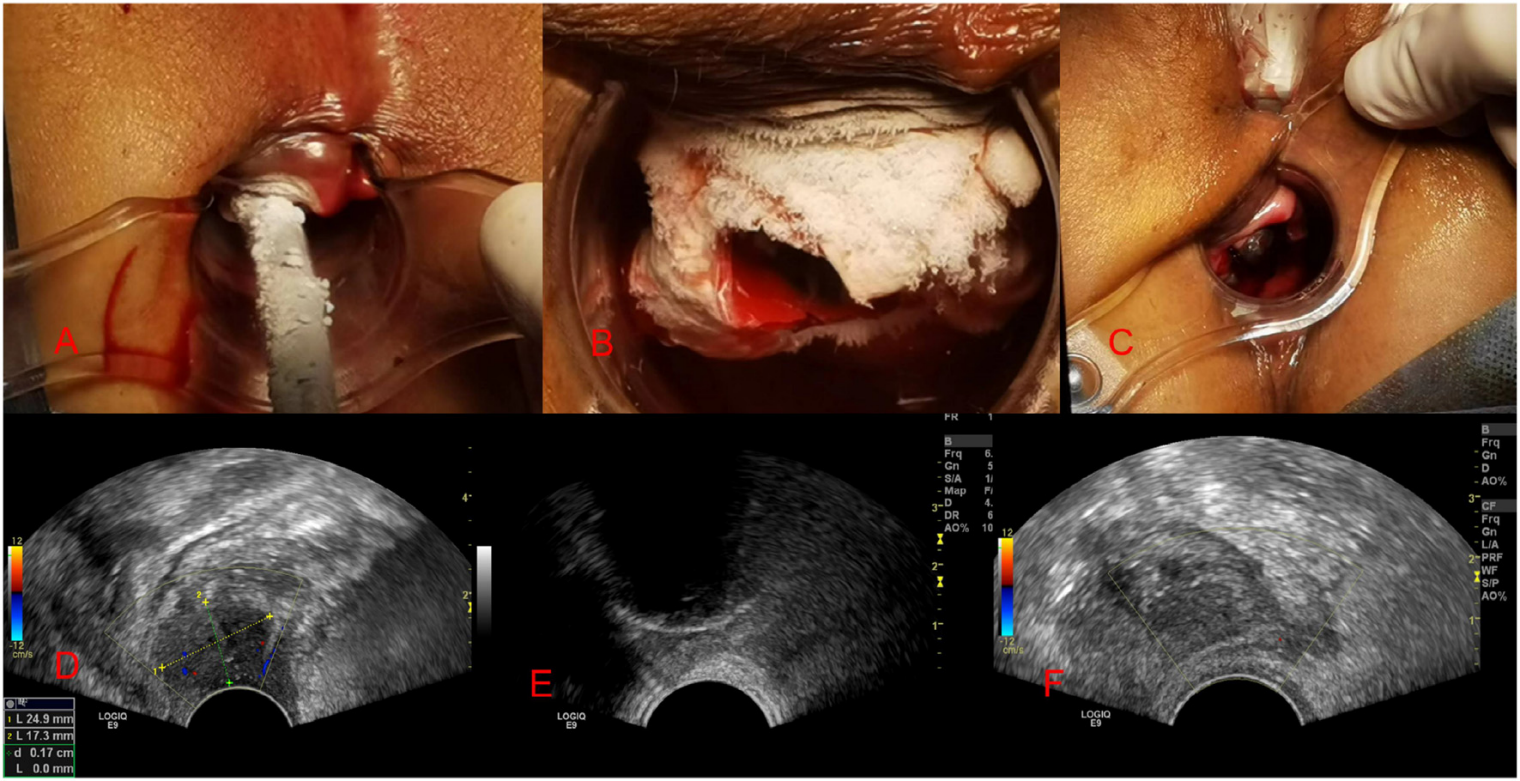
Images taken during cryotherapy and ultrasound monitoring. **(A-C)** shows images of the freezing process, while **(D, E)** displays images of ultrasound monitoring during the freezing procedure.

The image in **Figure 2A** depicted the refrigeration equipment during the cryoablation process, with the cryoprobe initiating its cooling effect. In **Figure 2B**, the tumor was enveloped by an ice ball, rendering it indiscernible to our sight. **Figure 2C** illustrated the ongoing transvaginal ultrasound monitoring following the initial freeze-thaw cycle. **Figure 2D** presented the tumor’s location and dimensions (2.4 × 1.7 cm) via ultrasound imaging prior to freezing. **Figure 2E** exhibited the progressive development of the ice ball, along with the heightened echogenicity at its perimeter. Finally, **Figure 2F** demonstrated the absence of tumor blood supply on contrast-enhanced ultrasound post-cryoablation.

## Post-cryoablation treatment

On the third day post-surgery, the patient experienced rectal bleeding that ceased without the need for intervention. No additional symptoms, such as fever, abdominal pain, or bloating, manifested following the cryoablation procedure. Following five days of antibiotic injections post-cryosurgery, the patient was discharged from our hospital. Subsequently, the frozen tumor underwent necrosis and falls off, resulting in a substantial ulcer in the rectum over the next half month. The ulcer was fully repaired within 1.5 months (**Table 1**).

**Table 1:** Timeline.

May, 2017	Hematochezia
June 13, 2017	Hospitalization treatment
June 21, 2017	MDT discussion
June 22, 2017	Cryosurgery
June 29, 2017	Leave hospital
July 15, 2017	PD-1 treatment per two weeks, 15 times in total.
Up to 2023	Outpatient follow-up until 2023
February 2024	Hematochezia again
March 18, 2024	Hospitalization treatment
March 22, 2024	Cryosurgery
March 27, 2024	She experienced a sudden cerebral infarction and started using Plavix anticoagulant therapy
Up to now	Rehabilitation treatment for cerebral infarction with occasional rectal bleeding

Two weeks post-discharge, the patient reported no discomfort. Given that cryotherapy is a localized ablation technique, a PD-1 inhibitor was administered based on the Multidisciplinary Team’s treatment recommendation to ensure therapeutic efficacy. Toripalimab (150mg per dose) was administered intravenously for immunotherapy biweekly, for a total of 15 treatments. Due to her advanced age, the patient ceased taking Toripalimab upon achieving treatment objectives. No serious adverse drug reactions were observed during the PD-1 treatment regimen. No radiotherapy, chemotherapy, or targeted therapy was administered to this patient due to her advanced age.

## Follow-up

Given the patient’s delicate age, we implemented an outpatient follow-up protocol post-discharge. Over a 5-year period of monitoring, we observed no signs of recurrence *in situ* (**Figure 1E**) nor any organ metastases. This confirmed her attainment of a 5-year survival milestone, with her disease-free survival period continuing to lengthen. Nonetheless, in the seventh year, the patient experienced rectal bleeding anew in February 2024. No additional symptoms were detected. A digital rectal examination revealed a tumor measuring 2 * 3cm on the right anterior wall of the rectum, close to the dentate line. Following imaging and pathological assessments, she was diagnosed with ARMM for the second time and subsequently underwent cryotherapy after multidisciplinary team (MDT) deliberations at the hospital. Five days subsequent to the procedure, the patient suffered an acute cerebral infarction and was treated with anticoagulant therapy using Plavix. Consequently, due to the presence of ulcers, the patient experienced intermittent minor rectal bleeding half a month post-cryosurgery. Owing to her frail health status, it was deemed inappropriate to administer PD-1 or other anti-tumor medications.

## Discussion

ARMM is an exceptionally rare and fatal disease, predominantly affecting the anal mucinous epithelium or rectal sinus dentate lines (14, 15). The pathological staging of primary anorectal mucosal melanoma adheres to the American Joint Commission on Cancer (AJCC) guidelines for skin melanoma, as there is no dedicated staging system for anorectal melanoma (16, 17). Regardless of the surgical approach, ARMM carries a poor prognosis (18).

Our research findings, including the preservation of the sphincter and the therapeutic efficacy, validate the benefits of cryoablation combined with adjuvant Toripalimab. This approach presents itself as a viable alternative for patients with anal rectal mucosal melanomas (ARMMs), particularly for those who are keen on maintaining anal sphincter function. Furthermore, our data indicates that the survival rate is comparable to the median survival time of 24 months previously reported (19). Additionally, it has been observed that the volume of tissue affected by cryoablation can surpass that of wide local resection (12). Unlike surgical resection, cryoablation can penetrate beyond the muscular layer, making it potentially more significant for patients with ARMMs. It is also noted that cryoablation can harness its abscopal effect through immune mechanisms (7). In essence, cryoablation not only eradicates tumor cells but also stimulates the immune system by releasing cytokines, suggesting that ARMM could be an appropriate indication for cryoablation treatment. However, further studies are required to substantiate its therapeutic efficacy. Importantly, no severe adverse events classified as grade 3 or higher according to the Clavien-Dindo classification have been reported, thus affirming the safety of this protocol. Ultrasound monitoring has been instrumental in preventing cryoablation risks and complications by providing real-time visualization of adjacent structures during the procedure. For instance, it can help to avoid complications such as a rectal vaginal fistula during cryosurgery. Noninvasive monitoring of cryosurgery is essential for accurately tracking the freezing process *in situ* and assessing postoperative outcomes (20). Ultrasound monitoring has been utilized in the surveillance of cryotherapy for various tumors (21–23).

It is advisable that all patients diagnosed with ARMMs undergo genetic testing, including for BRAF and CKIT mutations, prior to initiating treatment. This testing can help guide the classification of the disease and the development of protocols for potential targeted therapies (24). Following surgical intervention or cryoablation, the administration of immune checkpoint inhibitors remains essential to prevent organ metastases (25). Consequently, we opted to treat our patient with a combination of Toripalimab and cryosurgery to enhance the therapeutic outcome. Cryoablation not only has a stimulating effect on immune responses during the tumor ablation process (26–28) but also, when combined with Toripalimab, it can further amplify the efficacy of immunotherapy in the fight against cancer. Moreover, no significant adverse drug reactions associated with Toripalimab were observed in this case. This study underscores the safety and efficacy of the combined therapeutic regimen.

Up to now, we haven’t seen any application of cryotherapy with adjuvant PD-1 in ARMM. Hence, it is the first time to report this combination treatment approach. The result of this study confirmed that cryotherapy with adjuvant Toripalimab improved the 5-year survival rate. However, more studies are needed to confirm this result.It is worth noting that, investigating the use of immunotherapy agents in combination with cryoablation, is worthy of additional study.

In the subsequent follow-up, ARMM resurfaced in 2024. The patient chose to undergo cryoablation once more. Post-second cryoablation, there was no indication of a correlation between cryotherapy and cerebral infarction; however, as an invasive procedure, we must remain vigilant for potential rare complications in other organs, particularly in elderly individuals. The follow-up strategy involved a digital rectal examination every three months. It remains crucial to conduct imaging examinations, such as CT, MRI, or PET-CT, particularly when there is uncertainty during a digital rectal examination. For cases with indeterminate diagnoses, pathological examinations can be utilized to establish a definitive diagnosis.

There are a variety of treatment options for ARMM, encompassing endoscopy, surgery, chemotherapy, targeted therapy, and immunotherapy. It has been reported that the decision between local excision and APR should be made on a case-by-case basis (29). The combination of surgical interventions with effective systemic therapies has been demonstrated to be advantageous for patients with ARMMs (30). Immunotherapy drugs encompass those targeting cytotoxic T-lymphocyte associated protein 4 (such as Toripalimab, ipilimumab, and tramelimumab), targeting programmed cell death receptor 1 (PD-1; including nivolumab and pembrolizumab), and those targeting programmed death ligand 1 (PD-L1; such as atezolizumab, avelumab, and durvalumab) (31, 32). However, further studies are still required to substantiate this, as of now, it has demonstrated a significant advantage. No major adverse events occurred. The patient demonstrated good tolerance to our treatment. The approach employed proved effective and achieved our objectives, yet further study is required for validation. Related research will be conducted in the near future.

In summary, further investigation into cryotherapy for ARMMs is warranted. Ultrasound-guided trans-anal cryoablation, when combined with adjuvant Toripalimab, presents a novel therapeutic strategy for individuals afflicted with ARMMs, particularly for those desiring anal sphincter preservation. This treatment regimen has been demonstrated to be both safe and efficacious. The patient in question underwent a procedure with minimal anesthesia and surgical risks, coupled with a swift recovery, and has remained alive for more than five years. It would be beneficial to further examine the efficacy of combining immune checkpoint inhibitors with cryoablation in a larger cohort of ARMM patients to maintain sphincter function. Additionally, it is important to study the hematological indicators’ trends and the immune synergistic mechanisms of ultrasound-guided trans-anal cryoablation with adjuvant Toripalimab. Further definition of the relationship between the ice ball’s extent and tumor size, along with the clarification of indications and standardization of postoperative treatment protocols, remains imperative.

## Patient perspective

The patient remained eager to undergo cryoablation, signifying that the procedure has indeed conferred numerous benefits upon her, including the preservation of the anus, minimal trauma, and rapid recovery. From her viewpoint, she was content that no significant complications had arisen. Most importantly, cryoablation maintains her anal sphincter function, preventing the need for anal excision and substantially enhancing her quality of life.

## Data Availability

The original contributions presented in the study are included in the article/supplementary material. Further inquiries can be directed to the corresponding author.
